# Ellagic Acid Protects Cardiac Arrhythmias Following Global Cerebral Ischemia/Reperfusion Model

**DOI:** 10.31661/gmj.v8i0.1235

**Published:** 2019-02-25

**Authors:** Mahin Dianat, Khojasteh Hoseiny Nejad, Alireza Sarkaki, Yaghoub Farbood, Mohammad Badavi, Mohammad Kazem Gharib-Naseri

**Affiliations:** ^1^Physiology Research Center, Ahvaz Jundishapur University of Medical Sciences, Ahvaz, Iran; ^2^Department of Physiology, Faculty of Medicine, Ahvaz Jundishapur University of Medical Sciences, Ahvaz, Iran; ^3^Abadan Arvand International Division, Ahvaz Jundishapur University of Medical Sciences, Ahvaz, Iran

**Keywords:** Ellagic Acid, Ischemia/Reperfusion, Arrhythmias

## Abstract

**Background:**

Cerebral ischemia/reperfusion (I/R) could increase the reactive oxidative stress in the cardiomyocytes. Also, some studies report cardiac arrhythmias following oxidative stressor such as I/R. Hence, this study was aimed to investigate the effects of ellagic acid (EA) against arrhythmias in a cerebral I/R model.

**Materials and Methods:**

Thirty-two male rats were randomly allocated into four groups: Sham (normal saline, 10 days), EA (100 mg/kg EA, 10 days), I/R (20 min ischemia followed by 30 min reperfusion, 10 days), and EA + I/R (100 mg/kg EA before I/R). In all animals, electrocardiogram (ECG) was recorded pre-ischemia and postischemia on the first and 11th days, respectively.

**Results:**

The I/R group showed an abnormally prolonged QTc interval after ischemia compared to the preischemia and control groups. EA administration in the EA+I/R group significantly reduced this prolonged QTc interval (P< 0.01). In the I/R group, ischemic/reperfusion resulted in a prolonged QRS complex and an elevated ST, which EA significantly prevented (P<0.01). In addition, EA significantly prevented the dramatically shortened RR interval induced by reperfusion (P<0.01). The incidence of ventricular fibrillation significantly increased in the I/R group; then it dramatically decreased following the administration of EA (P<0.0001).

**Conclusion:**

EA pretreatment repaired the adverse effects of I/R on the ECG parameters, which can be attributed to its negative chronotropic effects. EA pretreatment can prevent the cerebral I/R-induced heart arrhythmias.

## Introduction

Cerebral ischemia/reperfusion (I/R) is the major cause of serious and long-term disability worldwide. Global cerebral ischemia arises when wide-spreading blood flow to the nervous system is severely disturbed, leading to a series of pathophysiological processes and the development of seizures [1]. Ischemic stroke is the most debilitating type of stroke, and global cerebral I/R has a high mortality rate of ischemia worldwide that can induce severe physical and neurological impairments and exacerbate heart failure [2]. Cardiomyocyte circadian clock dysfunction is linked to the pathogenesis of heart disease in response to stresses such as I/R [3]. Approximately 80%-85% of all stroke incidents are ischemic, caused by cerebral arterial thrombosis or embolism [4]. Several studies have demonstrated that inflammation plays a fundamental role in the pathophysiology of ischemic stroke [4-6]. A free-radical reaction has been reported as an important mechanism in brain damage [7]. Not only inflammation but also oxidative stress in I/R has been recognized as a reactive oxygen species (ROS), and they are the main mediators in pathological processes [8]. The brain consumes about 20% of the body’s total oxygen; however, it only makes up 2% of the body’s total mass. Therefore, it is very sensitive to oxidative stress [9]. Moreover, the brain contains large amounts of pro-oxidative metals and polyunsaturated lipids but with a very low antioxidant capacity. Therefore, the brain is very sensitive to the ROS system that promotes damage to not only several biomolecules but also the production of some inflammatory agents participating in the neuronal death process [9, 10]. Antioxidants are known as neuroprotective factors, reducing cerebral damage in I/R. In contrast to oxidative stress, antioxidants such as polyphenolic compounds have neuroprotective effects mainly by reducing the ROS [11, 12] and inducing cardioprotective effects [13]. Ellagic acid (EA) is a natural phenolic antioxidant present in several plant foods, which can reduce oxidative stress in I/R [14]. EA can increase the antioxidant enzymes such as catalase and glutathione in brain ischemia [15], and it can have a cardioprotective effect on chemical-induced arrhythmias in stress [16]. This study was aimed at investigating the protective effects of EA pretreatment on global cerebral I/R induced-cardiovascular injuries. The electrocardiogram (ECG) parameters were assessed comparatively pre-ischemia and postischemia in each group of rats, and the changes in the parameters were compared among the groups.

## Materials and Methods

### Animals

This was an experimental animal study conducted on 32 adult male Wistar rats (250-300 g) in the physiology department of Ahvaz Jundishapur University of Medical Sciences (AJUMS), Ahvaz, Iran. The rats were obtained from AJUMS’s animal house and were housed in four laboratory cages and maintained under the following conditions: a 12/12-hour light/dark cycle, sufficient access to food and water, constant temperature of 22±2 oC, and 50% humidity. They were maintained under these conditions for 14 days of acclimation. All the protocols were approved by the animal ethics committee of AJUMS (registry code: B-9348) and were in complete accordance with the National Academy of Sciences (eighth edition) guidelines.

### Global Cerebral I/R Induction

Global cerebral I/R inductionwas completed based on the procedures that were completely described in previous studies by Pulsinelli and Brierley [17], Hoseiny Nejad *et al*. [18], and Xue *et* al. [14]. After anesthesia, the rats underwent a temporary forebrain global ischemia [17]. On the second day, after 20 min of ischemia phase, reperfusion was started for 30 min with the opening of the carotid micro clamps. Similar procedures were performed for the sham and EA groups without global ischemia [14]. Serum samples were collected from blood samples obtained from the right ventricle through coagulation processes and 10-min centrifuging at 4000 rpm. Serum samples were then stored at -80°C.

### Study Design

The animals were randomly allocated into four groups (n=8 per group):

1. The Sham or negative control group received 100 mg/kg normal saline as a solvent for EA by oral gavage for 10 days.
2. The EA or positive group received 100 mg/kg of EA by oral gavage for 10 days.
3. The I/R group received the 1.5 ml/kg solvent (oral gavage, 10 days) and then underwent global cerebral I/R.
4. The EA+I/R group received 100 mg/kg of EA by oral gavage for 10 days and underwent global cerebral I/R.

### Recording ECG

Fifteen minutes after the anesthesia, a bipolar limb lead II electrocardiogram was recorded to detect heart rates (HR) and to assess the chronotropic effect, QTc interval (to assess the developing risk of ventricular arrhythmias), QRS voltage (to assess the inotropic effect), R-R interval (to assess the possibility of premature ventricular contractions), ST elevation, and ventricular fibrillation (VF) incidence (to assess the possibility of cardiac arrest). The ECG was recorded on the first and 11th days (before and after ischemia) by Bio-Amp and monitored using a Power Lab system (AD-Instruments, Australia) [16]. Bazett’s formula (QTc=QT/RR1/2) is widely used to calculate the corrected QT interval for human HR. The QT interval is related to the HR changes; short QT indicates an increased heart rate [19].All chemicals were purchased from Kimia-Zist Azma Company (Tehran, Iran).

### Statistical Analysis

The ECG variables were analyzed using a paired *t*-test and one-way analysis of variance (ANOVA), followed by the least significant difference (LSD) as a posthoc test for multiple comparisons. A P-value less than 0.05 was set at significant statistical.

## Results

### EA Effects OnQTc Interval After Ischemia

Comparison of QTc values before and after the ischemia in this study’s groups showedasignificant prolongation in each group (Table-1).Moreover, QTc values caused a significant difference in the EA+I/R rats compared with the I/R rats (P<0.05; F-value [3, 4]: 6). An abnormally prolonged QTc interval was observed after the ischemia in the I/R group compared with the preischemia condition in the control group (P<0.001; F-value [3, 4]: 11) but was significantly reduced by EA pretreatment in the EA+I/R rats.

### Effects of EA On ST Segment, R-R Interval, and QRS Duration Global Cerebral I/R Model

Comparison of the ST segment, R-R interval, and QRS duration induced by global cerebral I/R, showedsignificant differences in the studied groups (Table-2). Respectively, a prolonged QRS duration, a short R-R interval, and an elevated ST were observed in I/R and EA+I/R rats compared to the control groups. In addition, these changes caused a significant difference in EA+I/R rats compared to I/R rats (P<0.05; F-value [2, 3]: 6; Table-2).

### Effects of EA On the Incidence of VF Induced by Global Cerebral I/R

Comparison of the incidence of VF in various groups showed a significant increase in other groups compared to the control groups. There was not VF incidence in sham and EA groups, but it was 25% and 75% for I/R and EA+I/R, respectively, which showed a significant difference compared to the control groups (P=0.0005). Moreover, EA+I/R rats showed a reduced VF incident compared with the I/R rats (P< 0.01).

## Discussion

The results of this study demonstrated significant abnormal alterations in ECG variables from the I/R model, including a prolonged QTc interval, a prolonged QRS duration, a short R-R interval, an elevated ST, and an increased VF incidence in the experimental models. The EA pretreatment significantly prevented these I/R-induced abnormal changes of ECG parameters in I/R and EA+I/R groups. Boyuk *et al*. (2011) reported a significant therapeutic effect of EA on intestinal I/R injury in rats’ lung tissue [20]. Meerson and Belkina (1986) showed the protective effect of ionol as an antioxidant on ischemia, arrhythmias, and heart fibrillation in the male rats [21]. They reported that reperfusion could induce more severe ventricular arrhythmias than ischemia. Other types of arrhythmias, tachycardia, and extrasystole also occurred during I/R. The pre-administration of ionol eliminated VF during I/R. Ionol significantly reduced the incidence of tachycardia and extrasystole. The present study’s findings confirm the results of Meselson and Belkina’s study. EA, as an antioxidant agent like ionol, could significantly reduce VF incidence and arrhythmias during I/R. Seeram *et al*. (2005) reported EA’s antioxidant and antiproliferative properties in some in-vitro and small-animal models [22]. EA, as an antiproliferative antioxidant, could directly inhibit some carcinogens’ DNA binding [23, 24]. Kannan and Quine (2011) investigated EA’s cardioprotective effects on the myocardial infarction and reported that EA reduced the lipid peroxidation and levels of biochemical markers. Moreover, EA significantly increased antioxidant activity and content in the isoproterenol groups, which subsequently prevented oxidative stress in myocardial infarction [25]. In their follow-up study, Kannan and Quine (2013) demonstrated EA’s inhibitory effects on arrhythmias, hyperlipidemia, and hypertrophy during myocardial infarction in rats [26]. Despite many studies conducted on EA’s antioxidant, pharmacodynamics, and cardioprotective effects [27, 28], only a few of them have investigated the EA pretreatment’s effects on the global cerebral I/R model. One of the limitations of our study was that the laboratory biomarkers related to the global cerebral I/R were not evaluated.

## Conclusion

EA pretreatment could have beneficial effects on global cerebral I/R by altering I/R-induced adverse effects on ECG parameters. EA showed positive inotropic and negative chronotropic effects. In addition, EA pretreatment prevented the global cerebral I/R-induced heart arrhythmias.

## Conflict of Interest

The authors have no conflict of interest to declare.

**Table 1:** Comparisons of QTc Values Before and After Ischemia Among Studied Groups. Values Are Expressed as Mean ± SEM.

**Groups**	**QTc before I/R (milliseconds)**	**QTc after I/R (milliseconds)**	**P-value**	**F-value (3, 4)**
**Sham**	84.71 2 ±**.5**	84.5± 3	0.57	0.76
**EA**	88.42 ± 2**.5**	87.28± 2.**5**	0.56	0.78
**I/R**	86.28± 1.**5**	130 ± **2**	0.0007	11
**EA+IR**	87.14± 3.**5**	98.14± 3.**5**	0.01	6

**EA:** Ellagic acid; **I/R:** Ischemia/reperfusion

**Table 2:** Comparisons of QRS Duration, R-R Interval, and ST Elevation Between. Data Are Presented as Mean±SEM

**Groups**	**QRS duration** **(s)**	**R-R interval** **(s)**	**ST elevation** **(mV)**
**Sham**	0.0121 ± 0.0065	0.272 ± 0.021	0.088 ± 0.007
**EA**	0.0122 ± 0.0065	0.262 ± 0.0425	0.0946 ± 0.011
**I/R**	0.022 ± 0.06^a^	0.159 ± 0.066^a^	0.25 ± 0.0365^a^
**EA+IR**	0.0146 ± 0.0015^b^	0.234 ± 0.0055^b^	0.142 ± 0.4^b^

**EA:** Ellagic acid; **I/R:** Ischemia/reperfusion **a**P<0.001 vs. control groups; **b** P<0.05 vs. I/R group
